# Rapid carbon turnover beneath shrub and tree vegetation is associated with low soil carbon stocks at a subarctic treeline

**DOI:** 10.1111/gcb.12793

**Published:** 2015-02-18

**Authors:** Thomas C Parker, Jens-Arne Subke, Philip A Wookey

**Affiliations:** 1 Biological and Environmental Sciences, School of Natural Sciences, University of StirlingStirling, FK9 4LA, UK; 2 Department of Animal and Plant Sciences, Alfred Denny Building, University of SheffieldSheffield, S10 2TN, UK; 3 Environmental Sciences, School of Life Sciences, Heriot-Watt UniversityEdinburgh, EH14 4AS, UK

**Keywords:** *Betula*, carbon inventory, dwarf birch, ectomycorrhiza, gas flux, shrub expansion, soil carbon cycling, subarctic

## Abstract

Climate warming at high northern latitudes has caused substantial increases in plant productivity of tundra vegetation and an expansion of the range of deciduous shrub species. However significant the increase in carbon (C) contained within above-ground shrub biomass, it is modest in comparison with the amount of C stored in the soil in tundra ecosystems. Here, we use a ‘space-for-time’ approach to test the hypothesis that a shift from lower-productivity tundra heath to higher-productivity deciduous shrub vegetation in the sub-Arctic may lead to a loss of soil C that out-weighs the increase in above-ground shrub biomass. We further hypothesize that a shift from ericoid to ectomycorrhizal systems coincident with this vegetation change provides a mechanism for the loss of soil C. We sampled soil C stocks, soil surface CO_2_ flux rates and fungal growth rates along replicated natural transitions from birch forest (*Betula pubescens*), through deciduous shrub tundra (*Betula nana*) to tundra heaths (*Empetrum nigrum*) near Abisko, Swedish Lapland. We demonstrate that organic horizon soil organic C (SOC_org_) is significantly lower at shrub (2.98 ± 0.48 kg m^−2^) and forest (2.04 ± 0.25 kg m^−2^) plots than at heath plots (7.03 ± 0.79 kg m^−2^). Shrub vegetation had the highest respiration rates, suggesting that despite higher rates of C assimilation, C turnover was also very high and less C is sequestered in the ecosystem. Growth rates of fungal hyphae increased across the transition from heath to shrub, suggesting that the action of ectomycorrhizal symbionts in the scavenging of organically bound nutrients is an important pathway by which soil C is made available to microbial degradation. The expansion of deciduous shrubs onto potentially vulnerable arctic soils with large stores of C could therefore represent a significant positive feedback to the climate system.

## Introduction

Northern high latitudes, particularly north of 60° over land, and across the Arctic Ocean, have warmed by between 1–4 °C since 1960, and at a rate substantially greater than the global mean (Serreze & Francis, 2006; Hansen *et al*., 2010; Serreze & Barry, 2011). The ‘Arctic Amplification’ of global warming is also predicted to accelerate in the coming decades, further accentuating the contrasts with overall planetary warming (Serreze & Barry, 2011). In parallel with this strong warming trend, one important change in arctic and sub-arctic tundra ecosystems has been an increase in productivity (Guay *et al*., 2014) where some areas have experienced increases of up to 10 g phytomass m^−2^ yr^−1^ in the last 30 years (Epstein *et al*., 2012). Contributing towards productivity increase has been an expansion of the range of woody deciduous shrub species within the genera *Betula, Salix* and *Alnus* (Tape *et al*., 2006). Shrub range expansion has now been documented to be occurring at many sites across the Arctic at ecosystem (Myers-Smith *et al*., 2011) and plot scales (Elmendorf *et al*., 2012b). This concurs with changes predicted by warming experiments (Elmendorf *et al*., 2012a).

Plant-soil interactions play a key role in global biogeochemical cycles, modulating the fate of carbon (C) fixed by plants, and the amount stored in the soil (Heimann & Reichstein, 2008; Metcalfe *et al*., 2011). It is well-documented that supply of C to, and respiration from, the soil and roots is broadly proportional to primary productivity in the system (Litton *et al*., 2007; Chen *et al*., 2011; Metcalfe *et al*., 2011). However, although global scale analyses of the relationship between primary productivity and both plant and soil C stocks reveal general patterns (i.e., that the ratio of soil to vegetation C density increases with increasing latitude; Lal, 2005), they mask important local and regional contrasts associated with specific plant functional types and, for example, their mycorrhizal symbionts. Despite their obvious importance, these patterns and interactions are still not well-understood (Arneth *et al*., 2010; Van Groenigen *et al*., 2014).

In Northern terrestrial ecosystems, the expansion of woody species with more recalcitrant litter than the existing vegetation could lead to C sequestration in the soil and therefore a negative feedback to climate warming (Cornelissen *et al*., 2007). A birch forest in northern Scandinavia, for example, was found to contain more recalcitrant carbon compounds than adjacent ericaceous heaths (Sjögersten *et al*., 2003), which were suggested to be less prone to microbial decomposition. However, evidence is emerging that the supply of carbon via the rhizosphere of some woody species also stimulates decomposition of these recalcitrant (and potentially older) C stores (Hartley *et al*., 2012) in a process known as ‘positive priming’ (Kuzyakov, 2002). This, therefore, may shift the balance between productivity and respiration, resulting in low soil C sequestration in spite of high net primary productivity.

Empirical data from field studies is providing growing evidence that specific relationships exist between the vegetation type and biomass in arctic and boreal ecosystems and the amount of C stored in the soil (Wilmking *et al*., 2006; Kane & Vogel, 2009; Hartley *et al*., 2012). These do not conform to the positive relationships between productivity and C storage predicted by global C cycle models (Cramer *et al*., 2001; Qian *et al*., 2010; Todd-Brown *et al*., 2014). Arctic species’ below-ground biomass does not increase with Leaf Area Index (LAI) above 1 m^2^ m^−2^ (Sloan *et al*., 2013), and therefore may also defy predictions of carbon storage. At one site in northwest Alaska, Wilmking *et al*. (2006) revealed that recently advanced forest and shrub tundra had lower soil C densities in organic horizons than the adjacent tundra. Furthermore, Hartley *et al*. (2012) demonstrated that soil C densities in a Swedish sub-arctic forest were significantly lower than a nearby tundra heath. Kane & Vogel (2009) also showed that less C is stored in Alaskan boreal ecosystems where there is greater above-ground biomass. Taken together, these studies indicate that existing patterns of above- and below-ground biomass and C stocks along *spatial* vegetation transitions may hold clues regarding the possible consequences of *temporal* shifts in vegetation communities in the future (‘space-for-time substitution’). However, it is important to emphasize that C densities in many soils of the circumpolar north are often orders of magnitude higher than the phytomass in this region (Tarnocai *et al*., 2009; Hugelius *et al*., 2011; Epstein *et al*., 2012), and have developed over decades to millennia; this raises the prospect of northern ecosystems increasingly being at ‘dynamic disequilibrium’ (Luo & Weng, 2011) with contemporary climate.

There are a number of phenomena that could lead to a net loss of C from tundra ecosystems when shrubs and forests encroach. Firstly, there is a concurrent increase in the abundance of ectomycorrhizal (ECM) fungi with increasing cover by trees and shrubs. These fungi are one of the primary recipients of autotrophic C (Hobbie, 2006) and are able to produce and exude a number of structural carbon-degrading compounds (Cullings *et al*., 2008; Talbot *et al*., 2008). Although it is uncertain the extent to which these compounds may interact with soil organic carbon (SOC) in the Arctic, it is clearly of pressing importance to find out. Secondly, the input of ‘novel’ litter into the system (i.e., from plant functional types not previously substantial components of the community) could lead to faster C cycling if the nutrients are in forms more accessible to the decomposer communities, physically or biochemically, than the litter of the plants they are replacing (e.g., ericaceous species) (Read & Perez-Moreno, 2003). However, a replacement of graminoids (grasses and sedges) may lead to the opposite effect (Cornelissen *et al*., 2007). Thirdly, the accumulation of snow in drifts formed by taller vegetation and the resulting increased winter soil temperatures (Sturm *et al*., 2005) may lead to faster C turnover in winter (Schimel *et al*., 2004).

Other than the suggestion of ‘positive priming’ in subarctic birch forests, the ecological mechanisms by which C could be lost from the soil remain unresolved. Because the arctic tundra is undergoing increases in productivity (Epstein *et al*., 2012; Guay *et al*., 2014) on soils that contain a very substantial proportion of global soil C (Tarnocai *et al*., 2009), there is a compelling need to understand the process implications for rates of soil organic matter (SOM) turnover and both C sequestration and release.

The increase of woody shrub cover in arctic systems occurs over a gradient from low densities to dominance over time (Myers-Smith *et al*., 2011; Elmendorf *et al*., 2012b) and it is important to understand the effect on C storage of this more subtle change as well as the larger-scale differences between forest and tundra. The ecotone between forest and tundra merits sampling over spatial scales sufficiently fine-grained to underpin an improved mechanistic understanding of the relationship between plant cover, C fluxes and soil C stocks. At fine (nominally defined here as 1 to 100 m lateral) scales, such transitions include subtle but important elements such as a transitional shrub community. In this case the ‘space-for-time’ substitution also potentially matches likely successional changes (vegetation shifts) associated with climate change, albeit with changes in soil C stocks likely trailing changes in vegetation (Sistla & Schimel, 2013).

This present study of SOC stocks and ecosystem respiration across the forest-tundra ecotone makes use of a dispersed ‘mosaic-like’ treeline near Abisko, Sweden. The following hypotheses were tested:

1. In spite of higher productivity (Shaver, 2010), deciduous shrub and forest plots have lower soil organic horizon and total SOC than heath sites, likely due to higher decomposition rates;
2. At small scales at tundra heath sites, deciduous shrub cover is correlated negatively with SOC densities;
3. Shrub and forest plots have high rates of C recycling (respiration), which would be a key indicator of C loss from the ecosystem;
4. Ectomycorrhizal hyphal growth (a key link between plant productivity and soil C cycling) is comparable at shrub and forest sites, and both are higher than at heath sites.

## Materials and methods

### Sites description

Twelve independent, short (<100 m) transects were selected within a permafrost-free landscape (c 2 km^2^) spanning the subarctic/alpine treeline at Nissunsnuohkki (Abisko area, Sweden; ca. 68°18′N 18°49′ E, 600 m asl, hereafter referred to as ‘Abisko’). In this study, we adopt the terminology of Walker (2000) and Kaplan *et al*. (2003), presented in ACIA (2005), to distinguish tundra plant growth forms and to place the study into circumpolar context. The treeline is formed by mountain birch (*Betula pubescens* Ehrh. ssp *czerepanovii* (Orlova) Hämet Ahti) with an ericaceous understorey and typically moves through a thick layer of shrub vegetation (*Betula nana* L. and grey willow (*Salix*) species (Specifically, *Salix glauca*, often accompanied by *Salix lanata*; other *Salix* spp., including *S. hastata* and *S. lapponum*, occur less frequently) – before becoming tundra heath, dominated by *Empetrum nigrum* L. ssp *hermaphroditum* (Hagerup) Böcher and *Vaccinium vitis-idaea* L. This transitional shrub-dominated vegetation is similar to the ‘low- and high-shrub tundra’ (‘Continuous shrubland, 50 cm to 2 m tall, deciduous or evergreen, sometimes with tussock-forming graminoids and true mosses, bog mosses and lichens’) referred to in ACIA (2005), although generally not exceeding 1.5 m height and with the only one evergreen shrub species, *Juniperus communis* L., at low abundances. Tundra heath is here similar to the ‘erect dwarf-shrub tundra’ (‘Continuous shrubland 2 to 50 cm tall, deciduous or evergreen, with graminoids, true mosses and lichens’) of ACIA (2005). Soils in the forest are microspodosols with a thin O horizon (< 5 cm) underlain by glacial till on a bed-rock typically of hard-shale (Sjögersten & Wookey, 2002). Soil pH in the organic horizon is 4.3 ± 0.1 at forest and 4.5 ± 0.1 at heath locations in the Abisko area (Table 1).

**Table 1:** Vegetation characteristics along transects at Abisko across all blocks (means ± 1 SE, *n* = 12). ‘Canopy height’ refers to the actual vegetation canopy for Heath, Shrub-Heath and Shrub communities, and the understorey for the Forest Edge and Forest (where mountain birch trees comprise the canopy)

	Plot on transect				
	Heath	Shrub-Heath	Shrub	Forest Edge	Forest
Distance from Heath (m)	n/a	14.6 ± 1.6	28.3 ± 2.9	44.9 ± 5.8	67.6 ± 5.9
Canopy height (cm)	14.7 ± 0.7	21.2 ± 1.2	32.0 ± 2.4	27.9 ± 3.0	19.0 ± 1.7
*B. pubescens* density (trees m^−2^)				0.07 ± 0.01	0.07 ± 0.01
*B. nana* cover (%)	21.2 ± 2.7	36.9 ± 6.9	60.3 ± 4.8	32.2 ± 4.2	8.0 ± 2.2
*E. nigrum* cover (%)	65.4 ± 3.3	67.6 ± 3.4	66.9 ± 4.7	43.0 ± 6.5	45.4 ± 4.2
pH (organic horizon)	4.3 ± 0.1	4.6 ± 0.2	4.4 ± 0. 1	4.5 ± 0.1	4.5 ± 0.1

**Fig. 1:**
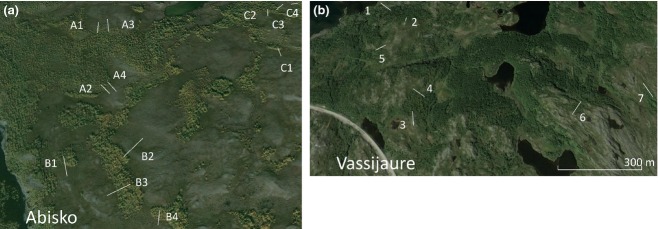
Google Earth images showing (a) Abisko transects and (b) Vassijaure transects across multiple treeline ecotones. At Abisko, A, B and C refer to different geographical blocks.

Transect lengths ranged from 52 to 97 m (Table S1) depending on the length-scale of the forest- heath ecotone. Care was taken to select vegetation transitions that were not present as a result of strong topographical influence; for example where water and snow accumulation due to dips and hollows dominate site conditions, and avoiding steep slopes [mean elevation change from heath to forest plots of −2.7 m (Table S1)]. Transects were selected with a variety of contrasting compass bearings (Table S1) to ensure that there was no bias in the data due to shading or winter snow drifting. The 12 transects were grouped geographically into three blocks of four as shown in Fig.1.

Seven further transects (over approximately the same area as the Abisko transects) were sampled at Vassijaure (68° 26′ N 18° 15′ E, 517 m asl). This location has monthly temperatures similar to the Abisko area (both monthly means range from −11.9 °C in January to 11 °C in July) but a far higher mean annual precipitation [848 mm compared with 304 mm; for an overview of environmental conditions at the two sites, see Sjögersten & Wookey (2005)]. Care was taken to distribute transects over an area similar in extent to Abisko, and to run transects over similar distances (c. 58 m). As with the Abisko sites, Vassijaure sites were selected to have little (on average) topographic change from H to F sites; this was, however, unavoidable for some sites (Table S1). Nevertheless, the most important apparent difference between sites was the vegetation community.

Five plots were established along each transect to represent best the transition in vegetation from heath to forest. These were; tundra heath (H), shrub heath (SH), shrub (S), forest edge (FE) and forest (F) (see Table 1 for further site details). H plots were chosen for an open heath environment with low *B. nana* cover and a low canopy height, and with vegetation dominated by *E. nigrum*. S plots were identified as areas dominated by *B. nana* with shrub height characteristically between 40 and 60 cm. SH plots were at locations intermediate between H and S plots, defined as having intermediate canopy height and *B. nana* cover, and generally located approximately equidistant to plots H and S. FE plots were located at the first *B. pubescens* tree along the transect from H to F and signified the forest margin. F plots were chosen to be in areas dominated by *B. pubescens,* approximately 10 to 15 m inside the forest edge.

### Vegetation surveys

Percentage cover of selected species was estimated at each plot on transects. Five 0.25 m^2^ quadrats were placed at each plot, one at the centre point and four more located 2.5 m from the centre point, every 90°, starting at a random bearing. In each quadrat, percentage cover of *B. nana* and *E. nigrum* was estimated by eye and the height of the tallest shoot was measured from ground level. Canopy height refers to actual canopy height at plots H, SH and S, and understorey canopy height at plots FE and F; at the latter two plot types *B. pubescens* forms the canopy (estimated to be 2–4 m vertically). Density of *B. pubescens* individuals >50 cm high was measured within a 5 m radius of the centre points of sites FE and F.

### Soil organic carbon (SOC) estimation

Soil organic carbon was measured at every plot (H, SH, S, FE and F) on all transects at Abisko and the H, S and F plots of transects at Vassijaure. Five soil cores were taken at 2 m from the central point at headings of 0, 72, 144, 216 and 288°. A two cm diameter soil corer was pushed (using a sharp knife inserted around the margin to cut fibrous materials, including roots, and to avoid compression) into the soil to a depth at which the corer could not be inserted any deeper (assuming that parent materials or large clasts were reached), and depth of organic and mineral horizons recorded. Subsamples of mineral and organic soil were collected and pooled for the five coring locations on each plot. Samples were homogenized, dried (80 °C for 48 h) and sieved through a 2 mm sieve. Soil organic matter (SOM) content for each pooled sample was determined by loss on ignition (LOI) in a furnace at 550 °C for 5 h (Ball, 1964).

Bulk density (BD) was sampled once at the organic horizon at the centre point of every plot by vertically inserting a 6.5 cm diameter, 10 cm deep PVC collar, measuring depth of organic horizon in the collar and calculating volume of soil present. Bulk density samples were dried at 80 °C for 48 h (to ‘constant weight’) before determining soil dry mass. Five transects were selected to measure BD of mineral horizons. The procedure was the same as for the organic horizon except that this was removed to expose the mineral horizon. BD of mineral horizons across all sites and transects was found to be very consistent (1.20 ± 0.067 g cm^−3^; mean ± one standard error) therefore the mean bulk density across sites was applied to all mineral horizons in the calculation of SOM.

Soil organic matter content (kg m^−2^) in organic and mineral soil was calculated according to


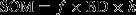


where *f* is the fraction of organic matter, *BD* the bulk density (kg m^−3^), and *h* the height of the respective horizons (m; averaged across the 5 cores).

Soil organic carbon was measured from all soil samples taken from Vassijaure (organic and mineral; H, S, F). Triplicate subsamples from each sample were measured for C content after combustion in a Vario EL Cube elemental analyser (Elementar, Hanau, Germany) and a mean was taken for each plot. The relationship between measured SOM (g g^−1^) and SOC (g g^−1^) was determined. Based on these samples, SOC can be calculated with high confidence (*P* < 0.001, *R*^2^ = 0.997) according to


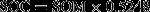


This equation was applied to estimations of SOM at every plot to estimate SOC.

### Respiration measurement

At all plots of the 12 Abisko transects, PVC collars with a diameter of 15 cm and a height of 7 cm were placed on the soil surface and sealed to the soil using a nonsetting putty (Plumber's Mait®, Bostik Ltd, Stafford, UK). Collars were not pushed into the soil to avoid disturbing the rhizosphere. Effectiveness of the seal was confirmed as all measurements of respiration showed a linear and regular increase in (CO_2_) which was comparable to closed system in laboratory conditions.

A portable EGM-4 infrared gas analyser with a darkened CPY-2 chamber (PP Systems International, Amesbury, MA, USA) was used to measure respiration. Respiration in this study is defined as the sum of microbial, root and shoot (including cryptogam) respiration within the chamber. At H plots, this measurement includes the entire vegetation canopy and therefore represents ecosystem respiration (ER); however, at all other sites the vegetation canopy is higher than the chamber, and the respiration measurement is therefore the sum of the understorey shoot and cryptogam respiration, total root and microbial respiration. CO_2_ flux was measured from all collars in June and September 2012 and June, July and September 2013. Respiration rates were calculated as the product of a linear function of CO_2_ concentration increase within the closed system, over a period of 90 s. Tests with longer regression periods showed no improvement of fit compared with regression results obtained over 90 seconds. All collars on every transect at Abisko (60 collars in total) were measured over periods of 2 days from 09:00–16:00 hours. Complete blocks were measured on the same days to avoid bias from variations in temperature and moisture over the 2 day periods. The order in which blocks and transects within blocks were measured was randomized, as was the order of sampling within transects (i.e., H to F or F to H).

### Hyphal in-growth

Thirty-seven μm nylon mesh bags (5 × 4 cm) were filled with 25 g sand from the shore of Lake Torneträsk (68°21′N, 18°49′E). No plants were present above-ground within 1 m of the sampling point. Sand was sieved to between 0.125 and 1 mm, rinsed under a flow of water for 1 minute then microwaved in a microwave (800 W) for 12 min, reaching a temperature of 98 °C. This process was repeated and rinsed a final time before drying for 48 h at 80 °C. Bags were inserted within 0.5 m of the PVC collar at the centre of the plots. The bags were left in the field for 92 days between 16th June and 16th September 2013. Sand was removed from the mesh bags and freeze-dried using a ModulyoD freeze drier (ThermoFisher Scientific, Waltham, MA, USA) for 72 h within 6 h of recovery.

One gram of sand from each bag was sonicated for 10 min in 30 ml of H_2_O, a 4 ml aliquot of the solution was filtered onto a nitrate cellulose filter paper using a Millipore filtration kit, and fungal material was stained with trypan blue. Hyphal length was counted under 200x magnification (Primo Star, Zeiss, Oberkochen, Germany) using the line intersect method (Brundett *et al*., 1994). This was repeated to make duplicates for each in-growth bag, a mean of which was taken as the final measurement.

### Defoliation event

In 2012 and 2013, there was a significant joint outbreak of the geometrid moths *Operophtera brumata* and *Epirrita autumnata* across the Abisko and Vassijaure areas, causing large scale defoliation the *B. pubescens* canopy and damaging the understorey. In a separate study at these sites, complete defoliation was observed to reduce respiration rates but only at 50 cm from the base of a tree, there was no significant effect of defoliation on soil CO_2_ flux further away from the tree (Parker *et al.,* Unpublished data). In the present study, all collars for respiration measurement are at least 2 m from the closest tree and therefore, we do not consider defoliation to have affected respiration rates significantly. ECM in-growth into sand was reduced by *B. pubescens* defoliation (average F and FE plot defoliated by 50.5%) by an average of 26.6% (Parker *et al*., Unpublished data). Therefore, the results presented in the present study in F and FE plots will likely be an underestimation compared to a ‘healthy’ year. At our plots the outbreaks were confined to the forests and there was no evidence of defoliation of H, SH or S plots.

### Statistical analysis

Differences in organic horizon SOC, mineral horizon SOC and total SOC between vegetation types, within sites (Abisko or Vassijaure), were analysed using one-way anovas. If the raw data did not meet the assumptions of parametric analysis, they were transformed using a natural log. If vegetation type was statistically significantly related to SOC, differences between vegetation types were analysed using a Tukey's Honestly Significant Differences (HSD) test. A generalized linear model, following Poisson distribution and a log-link function, was used to analyse the relationship between *B. nana* cover and organic horizon SOC. Repeated measures nested anovas following a linear mixed effects model were used to analyse for differences in respiration rates between vegetation types. A nested anova following a linear mixed effects model was used to analyse hyphal in-growth between vegetation types. The respiration and hyphal in-growth data were nested within transect then block, which were assigned as random factors. Respiration and hyphal growth data were square root transformed prior to analysis to meet the assumptions of the parametric model. Differences between vegetation types as analysed by nested anovas were identified using one degree of freedom Wald tests. All analyses were carried out on R studio v0.97.551.

## Results

### Soil organic carbon across ecotones

**Fig. 2:**
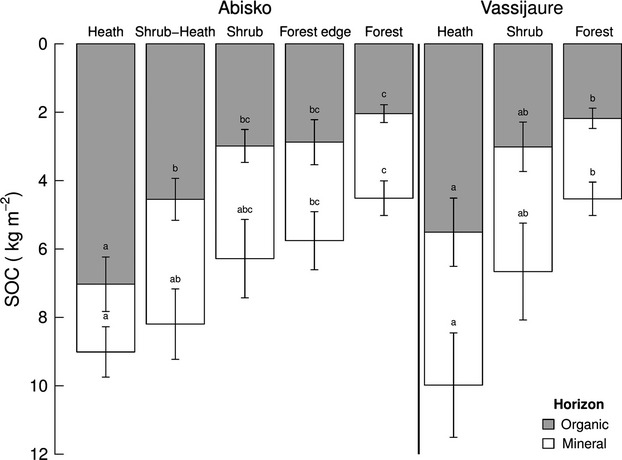
SOC at Abisko (dry/mesic, *n* = 12) and Vassijaure (mesic/wet, *n* = 7) across multiple heath-forest ecotones. The lower error bars (± 1SE mean) refer to total SOC (Organic + Mineral). The upper error bars (± 1SE mean) refer to organic horizon only SOC. Different letters show significant differences between means (*P* < 0.05) from Tukey HSD post-hoc tests (see Table 2 for test statistics). Letters refer to differences within site and horizon (Organic or Total).

**Fig. 3:**
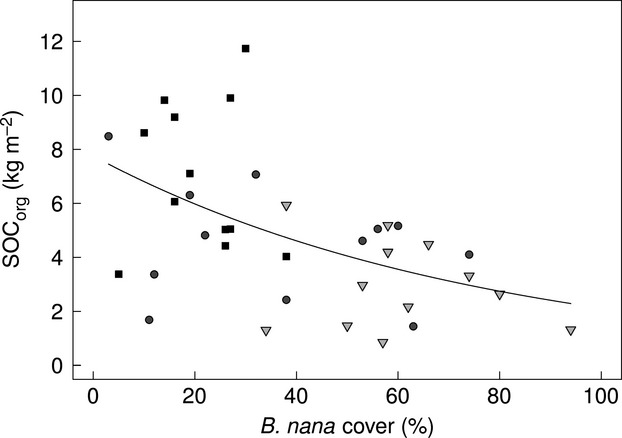
Relationship between % cover of *Betula nana* and SOC of the organic horizon at H (squares), SH (circles) and S (triangles) sites (*y* = 10^2.04 - 0.013x^). Modelled line represents a significant relationship between the two variables [generalized linear model (Poisson distribution, z = −3.722, *P* < 0.001, d.f. = 35)]

At Abisko there are significant differences in organic horizon SOC (SOC_org_) between vegetation types (Fig.2, Table 2 for statistics). Both S [2.98 ± 0.48 kg m^−2^ (mean ± 1SE)] and F (2.04 ± 0.25 kg m^−2^) plots have significantly lower SOC_org_ than the H plots (7.03 ±0.79 kg m^−2^) but are not significantly different from each other. Differences can be observed in SOC_org_ between H (7.03 ± 0.79 kg m^−2^) and SH (4.55 ±0.61 kg m^−2^) where *B. nana* cover increases by an average of 15.7% across an average lateral distance of 14.6 ± 1.6 m (Table 1). Furthermore, there is a significant (*P* < 0.001, Fig.3) negative relationship between the % cover of *B. nana* and SOC_org_.

**Table 2:** Test statistics for one way anovas analysing differences in organic horizon SOC (SOC_org_), mineral horizon SOC (SOC_min_) and total SOC (SOC_tot_) between vegetation types within sites (Abisko and Vassijaure). Data marked ‘^*^’ have been natural log transformed for analysis

	Abisko			Vassijaure		
Site	*F* value	d.f.	*P* value	*F* value	d.f.	*P* value
SOC_org_	11.18	4,55	<0.001	5.60	2,18	0.01
SOC_min_	0.66^*^	4,55	0.62	1.76	2,18	0.2
Total	6.38^*^	4,55	<0.001	4.94	2,18	0.02

At Vassijaure there is a significant relationship between vegetation type and SOC_org_ with a significant difference between H (5.51 ± 1 kg m^−2^) and F plots (2.18 ± 0.29 kg m^−2^) (Fig.2, Table 2). The difference in SOC_org_ between H and S (3.01 ± 0.72 kg m^−2^) was not as pronounced at Vassijaure as at Abisko and was not statistically different (*P* = 0.066). At both Abisko and Vassijaure there are no significant differences in mineral SOC (SOC_min_) between vegetation types (Fig.2, Table 2). Reflecting this, total SOC (SOC_tot_) follows a similar pattern to SOC_org_ across the vegetation types and at both sites, with a decrease in SOC_tot_ from H to F. There is a significant relationship between vegetation type and SOC_tot_ at Abisko (Fig.2, Table 2), with SOC_tot_ reducing from 9.01 ± 0.74 kg m^−2^ at H plots to 4.51 ± 0.51 kg m^−2^ at F plots. The first significant reduction in SOC_tot_ compared to H plots was at the FE plots (5.76 ± 0.84 kg m^−2^). As with SOC_org_, SOC_tot_ at Vassijaure follows a very similar pattern (Fig.2). In this case, the differences in SOC_tot_ between H (9.98 ± 1.53 kg m^−2^) and F (4.53 ± 0.49 kg m^−2^) plots are statistically significant (*P* = 0.016).

### Respiration rates at Abisko ecotones

**Fig. 4:**
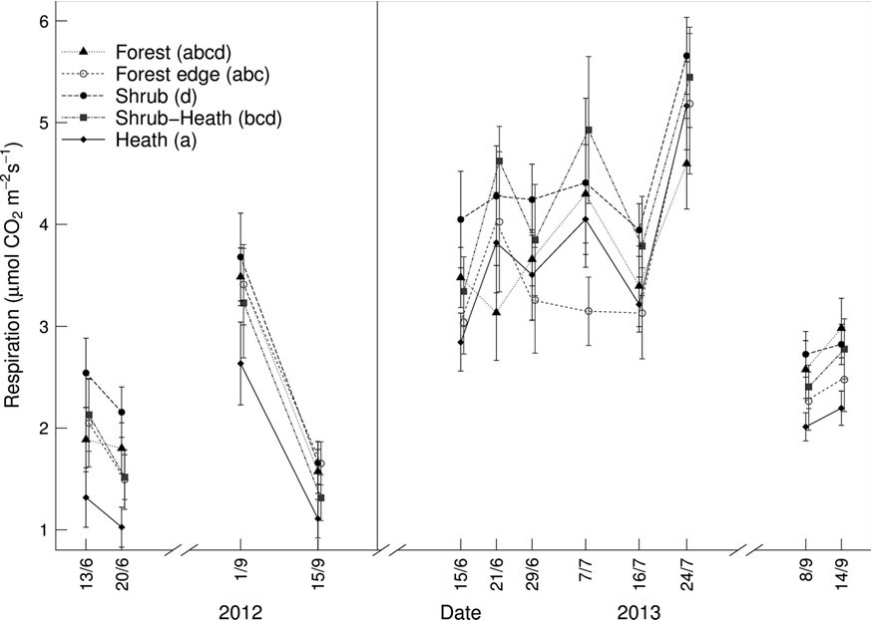
Dark respiration over two years of measurement across five vegetation types (*n* = 12). Repeated measures nested anova:*F* = 3.92, *P* = 0.0083, response variable was square root transformed before analysis to meet assumptions of the linear model. Different letters in brackets at the figure legend represent significant differences (*P* < 0.05) between vegetation types within the statistical model using one degree of freedom Wald tests.

**Fig. 5:**
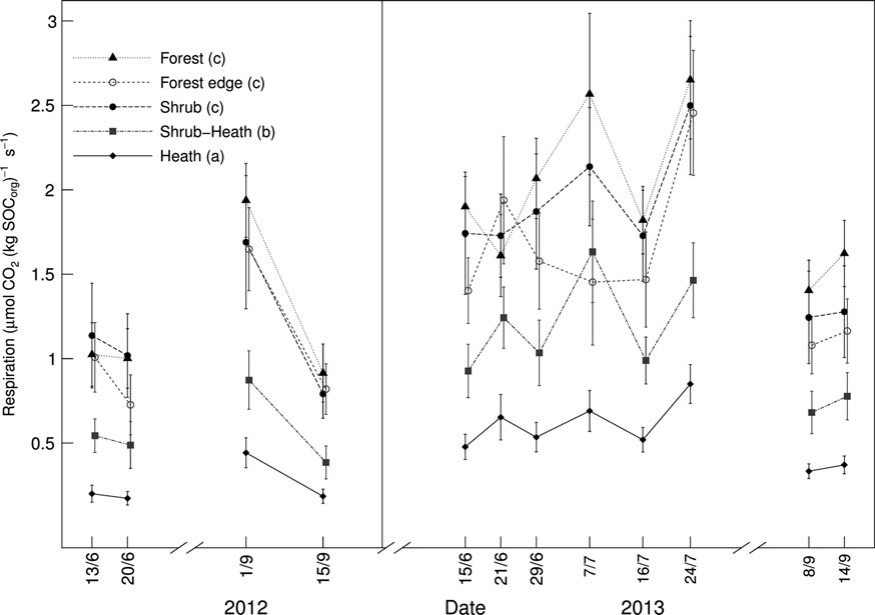
Dark respiration (expressed per kg SOC at each plot) measured over 2 years at five vegetation types (*n* = 12). Repeated measures nested anova:*F* = 12.90, *P* < 0.001, response variable was square root transformed before analysis to meet assumptions of the linear model. Different letters in brackets at the figure legend represent significant differences (*P* < 0.05) between vegetation types within the statistical model using one degree of freedom Wald tests.

Respiration was significantly (*P* = 0.008) associated with vegetation type (Fig.4). Mean respiration over all measurement points was highest at shrub plots (3.49 ± 0.21 μmol CO_2_ m^−2^ s^−1^), followed, in decreasing order, by SH, F, FE and H plots (3.23 ± 0.20, 3.03 ± 0.22, 2.93 ± 0.32 and 2.71 ± 0.13 μmol CO_2_ m^−2^ s^−1^, respectively); only the latter (H) was significantly different from S plots (p < 0.001). When respiration is expressed per kg SOC_org_, however, it was significantly associated with vegetation type (*P* < 0.001, Fig.5); S, FE and F plots respired at very similarly high rates (1.37 ± 0.29, 1.44 ± 0.22, 1.48 ± 0.19 *μ*mol CO_2_ (kg SOC_org_) ^−1^ s^−1^, respectively), followed by SH and H plots (0.77 ± 0.15 and 0.48 ± 0.08 μmol CO_2_ (kg SOC_org_) ^−1^ s^−1^, respectively), which were significantly lower (*P* < 0.001).

### Hyphal in-growth at Abisko ecotones

Hyphal in-growth increased steadily along the transect from H [6.79 m hyphae (g sand) ^−1^] to FE plots [17.70 m hyphae (g sand) ^−1^] with more hyphal growth in S (*P* = 0.018) and FE (*P* = 0.01) plots than H plots. There were lower growth rates at the F plots with a decrease to 10.67 m hyphae (g sand)^−1^ from the FE plots but this difference was not significant (*P* = 0.14). Likewise, the overall pattern was not statistically significant as indicated by the nested anova (*P* = 0.077).

## Discussion

Our results provide strong evidence to support a number of hypotheses relating to vegetation cover and C storage in the soil. First, they demonstrate, using 17 independently replicated transects over two landscapes, that SOC stocks are similar in deciduous shrub-dominated systems and forest systems, but substantially lower than in adjacent, lower productivity, tundra heath systems (Hypothesis 1). Our data show that this is true at multiple scales, from negative relationships between cover of *B. nana* and SOC (Hypothesis 2), to changes in SOC over ecotones. This emphasizes a close link between the dominance of nonericaceous woody species present in a community and the amount of C stored in the soil. We have shown that the changes in SOC over ecotones hold true at the landscape scale (both ca. 2 km^2^ sampling areas), and also are similar in contrasting climatic contexts (sites with large differences in mean annual precipitation).

Until now, only Wilmking *et al*. (2006) had shown that SOC is depleted in shrub tundra compared to tussock tundra over permafrost in NW Alaska. Our sites are not underlain by permafrost, and they are relatively freely draining; moisture and thermal status, alone, are therefore unlikely to explain contrasting rates of organic matter decomposition in shrub and forest communities compared with tundra heaths. Previous work at Abisko (Hartley *et al*., 2012) showed that SOC densities in subarctic birch forests were lower than at tundra heaths. They did not, however, consider other woody vegetation (specifically, nonericaceous shrub-dominated communities) in the same landscape; neither the ecological similarity between forest and shrub-dominated systems nor whether they exert the same controls over SOC and how it is cycled. Furthermore, our study reveals a fine-scale negative relationship measured between *B. nana* cover and SOC (Hypothesis 2). This may be important in predicting how ecosystems will respond to gradual vegetation change as observed at plot scales (Elmendorf *et al*., 2012b) and in warming experiments (Elmendorf *et al*., 2012a).

Root biomass is an especially important component of C storage in arctic ecosystems which in most cases is larger than aboveground biomass (Iversen *et al*., 2014). However, a full inventory of root biomass was beyond the scope of this study, but Hartley *et al*. (2012) provide data to indicate that it represents from ca. 8% to 18% of total below-ground C stocks in nearby heath and forest plots, respectively, in Abisko. Furthermore, at such sites, fine root C does not increase linearly with LAI above 1 m^2^ m^−2^ (it tends to plateau at approximately 0.25 kg C m^−2^) (Sloan *et al*., 2013). This suggests that extra C sequestered in above-ground biomass may not be associated with a proportional increase in root biomass. The mechanism for this is high root turnover at high LAI meaning that high production of roots in more productive vegetation types does not result increased storage of C in root biomass (Sloan *et al*., 2013). However, this has not been found for course roots in tundra (Campioli *et al*., 2009) or forest systems (Bolte *et al*., 2004).

We present data to show that the small C stocks under forest and deciduous shrub vegetation are being recycled (respired) substantially faster than adjacent, more SOC-rich, ericaceous heaths (Hypothesis 3). When the flux data are standardized and presented per unit SOC (i.e. potentially available substrate) present at each plot, it becomes clear that plots with high productivity (shrubs and trees) also return C rapidly to the atmosphere via respiration compared to adjacent tundra heath communities (Fig.5). Even without standardizing the respiration data per kg SOC_org_ we show that respiration is highest in deciduous shrub vegetation (Fig.4). Although we did not measure photosynthesis, previous work shows that photosynthetic rates can be up to five times higher in deciduous shrub vegetation compared to tundra heath (Shaver, 2010; but note that Fletcher *et al*. (2012) also provide evidence of some depression in rates of GPP per unit leaf area in transition zones compared with adjacent ‘main’ vegetation types). So, whilst we could not quantify all components of the C fluxes and stocks across our vegetation transitions, we show that the larger amounts of C that are likely assimilated into deciduous shrub plots compared to heath plots are quickly metabolized and returned to the atmosphere through respiration.

Our findings suggest that the increased amount of C fixed by shrubs is cycled at a faster rate and therefore not sequestered in the soil to the same extent as predicted by some models (Qian *et al*., 2010; Todd-Brown *et al*., 2014). Our data are consistent with measurements at other shrub sites with relatively warm soils, which have been shown to be slight net sources of CO_2_ (Cahoon *et al*., 2012). These authors concur that a shift to shrub dominance in the Arctic will increase rates of C cycling and result in loss of C to the atmosphere if temperatures continue to increase. At another site in the low Arctic of Northwest Territories, Canada, a warming experiment with strong increases in shrub productivity yielded no extra standing above-ground litter compared to control (Zamin *et al*., 2014), suggesting that the increase in productivity is concurrent with faster recycling and release of C from the ecosystem.

Here, we studied replicated vegetation transitions, thought to represent a plausible space-for-time scenario, to understand better the patterns that exist between vegetation and soil C and the possible future of soil C under vegetation change. The future flux of C is, however, highly dependent on a large number of interacting biotic and abiotic factors, several of which we have not investigated directly. The vegetation of arctic tundra can be highly heterogeneous over small spatial scales (Walker *et al*., 2005), and contrasting vegetation types can have significantly different fluxes of C. Moist sedge tundra, for example, is far more productive, with faster rates of C cycling, than adjacent dry heaths (Kade *et al*., 2012). Increased shrub abundance may therefore have contrasting effects on sedge tundra than on ericaceous heaths. Additionally, where carbon cycling is slow due (topographically) to waterlogged conditions (Zona *et al*., 2011), shrub vegetation may have a less pronounced effect on SOM decomposition due to the relatively greater importance of physico-chemical constraints (e.g., anoxia) on microbial activity. Shrubs have, in fact, been observed to increase in wet soils that have experienced climate warming (Elmendorf *et al*., 2012b), but a key question is by how much they will influence rates of C cycling once established.

We see that our observations hold true in both areas of very high and low rainfall (Sjögersten & Wookey, 2005) in the sub-Arctic, i.e., at geographical scales for which forest and shrub expansion have been observed in the Fennoscandian sub-Arctic (Tømmervik *et al*., 2009; Rundqvist *et al*., 2011). This gives us greater confidence that the hypothesized ‘vegetation effect’ we observe can be extrapolated over larger areas with contrasting climates. We also hypothesize that expansions of shrubs and trees across the sub- and low Arctic tundra, the majority of which is underlain by permafrost (Tarnocai *et al*., 2009), may result in net losses of SOC from organic horizons which are supplemental to changes caused by climate drivers (e.g., soil warming and drying, and active layer deepening). The patterns that we observe in our study should be applicable in continuous permafrost regions where shrub expansions (Myers-Smith *et al*., 2011) and productivity increases (Epstein *et al*., 2012) are occurring. Indeed, Wilmking *et al*. (2006) observed similar decreases in stocks of C in a permafrost-underlain region. It is therefore likely that shrub expansion in tundra that is underlain by permafrost will result in loss of SOC.

**Fig. 6:**
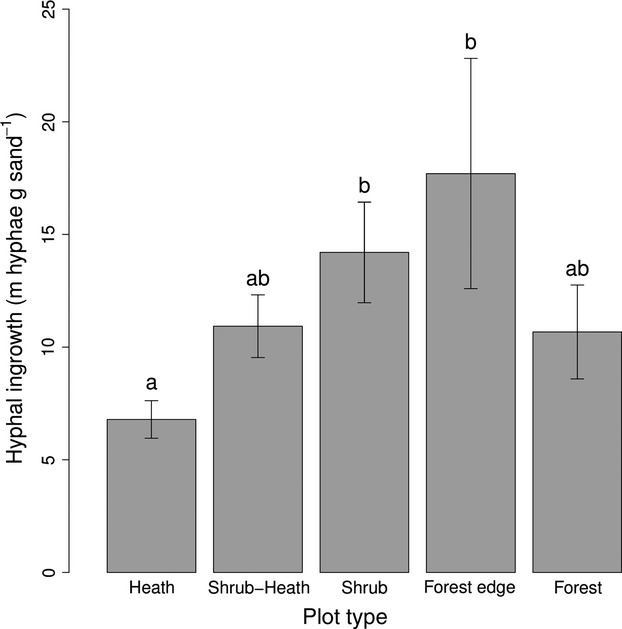
Hyphal in-growth of fungi over summer 2013 at Abisko transects. Nested anova:*F* = 2.28, *P* = 0.077, response variable was square root transformed before analysis to meet assumptions of the linear model. Different letters represent significant differences (*P* < 0.05) between vegetation types within the statistical model using one degree of freedom Wald tests.

Our results suggest that because there are similarly low SOC stocks in shrub and forest vegetation, there may be similar plant-soil interactions at work. One of the likely key differences between forest and shrub systems and tundra heaths at our study sites is the dominance of ECMs (Read & Perez-Moreno, 2003) in symbiosis with *B. nana* and *B. pubescens,* amongst others (Hypothesis 4). We have some evidence to support this, as we find that there is a general increase in ECM growth along the transects from the heath to the edge of the forest. The decrease seen at F plots in Fig.6 is likely to be due to the partial defoliation of some F and FE plots which will have reduced C flow to the ECM community and reduced hyphal growth (Parker *et al.,* Unpublished data) amongst other ECM community changes (Kuikka *et al*., 2003). This would act to dampen the effect that we observed (Fig.6) and we expect that FE and F plots have higher ECM growth rates in non-out-break years).

There is increasing evidence that the action of ECM fungi in scavenging for nutrients results in the breakdown of SOC (Talbot *et al*., 2008). The exact mechanisms for this are attracting considerable interest, and the relative importance of ECMs’ potential saprotrophic ability, their influence as an ‘accidental decomposer’, and as a direct recipient of plant C for positive priming, will be important to know (Talbot *et al*., 2008). One could view the transition from heath to shrub to forest as an increase in dominance of ECM fungi from heath to shrub vegetation, and then a plateauing at the forest which would explain the loss of SOC along this transition if the ‘decomposers in disguise’ hypothesis is true (Talbot *et al*., 2008).

Hartley *et al*. (2012) showed, by radiocarbon analysis of respired CO_2_, that ‘old’ SOC was being decomposed at peak growing season in a subarctic birch forest. They attributed this to recently assimilated (‘young’) C by the trees causing a positive priming effect (Kuzyakov, 2002); we propose here that the ECM community is central to this process. Ectomycorrhizals receive up to 20% of total C fixed by trees (Hobbie, 2006) and are therefore a key interface between labile C input and C sequestered in the soil. Ectomycorrhizals have substantial potential to produce extracellular enzymes to break down a range of structural organic compounds (Cullings *et al*., 2008; Talbot *et al*., 2008; Phillips *et al*., 2014). One such genus (*Cortinarius*) has been found to excrete SOC-targeting peroxidases in response to low nitrogen (N) availability in the soil in the same region as the present study (Bödeker *et al*., 2014). This finding may be of key importance in heath systems with relatively high soil organic C contents (e.g., H and SH plots), which typically also have low N availability (Read & Perez-Moreno, 2003), as the ECMs may degrade soil C to mineralize N (Bödeker *et al*., 2014). The observation in the current study that areas of high above-ground productivity and ECM growth (Fig.6) (Hypothesis 4) have the highest rates of C cycling (Hypothesis 3) and lowest SOC (Hypothesis 1) lends support to the hypothesis that the ECM symbiosis is a mechanism by which C is lost from the soil. This could be important following an expansion of vegetation with ECM associations into heath soils where nutrients such as nitrogen are more likely to be bound in organic forms (Read & Perez-Moreno, 2003).

One other mechanism that could explain, or contribute towards, the patterns in SOC that we have observed is the influence of winter processes. Over winter, an insulating layer of snow is trapped by shrubs and trees (Sturm *et al*., 2005), which contrasts with heath sites where drifting elsewhere results in only thin or no snow cover. This insulating snow layer may maintain a more active microbial community (Schimel *et al*., 2004) with higher winter respiration rates (Sullivan, 2010), which we propose could also contribute to the loss of SOC from the system. As with the ECM example, the pattern in SOC across the transect will be mirrored by a similar pattern in abiotic constraints over biogeochemical processes such as snow accumulation.

Lastly, the transition in vegetation from heath to forest represents a transition in chemical composition of litter input; there is a reduction in chemical recalcitrance and decomposability of litter from heath (evergreen dominated) to forest (deciduous dominated). *Empetrum nigrum* leaf litter has high concentrations of phenolic compounds, which results in low decomposition and accumulation of SOC (Tybirk *et al*., 2000). This contrasts with deciduous shrubs and trees (Cornelissen *et al*., 2007; Cornwell *et al*., 2008) and specifically *B. nana*, which decomposes faster than *E. nigrum* (Aerts *et al*., 2006). At our sites, there is a substantial cover of *E. nigrum* in the understory of the forest and shrub plots (Table 1), yet we do not observe accumulation of SOC at these plots. It is therefore likely that the chemical composition of the litter input is not the most important determinate of SOC storage at these plots. Much like the decomposition of *B. pubescens* litter (Sjögersten & Wookey, 2004), we hypothesize that decomposition of *E. nigrum* litter (amongst other litter types) is enhanced in shrub and forests systems due in part to the presence of a strong decomposing fungal community (Lindahl *et al*. 2007; Bödeker *et al*., 2014).

In conclusion, we present evidence for a marked contrast in below-ground C cycling rates across the forest-tundra ecotone at a subarctic treeline. Our results, based on a fully replicated design and covering contrasting landscape settings, not only confirm that mountain birch forests have relatively low soil C densities, but also that shrub vegetation has equally low SOC storage and faster C turnover. This relationship holds across different microclimatic conditions (contrasting precipitation at comparable mean temperatures), supporting the hypothesis that treeline vegetation type strongly controls SOC storage. These data emphasize the importance of plant-soil interactions and of the relative size, responsiveness and vulnerability of phytomass and SOC stocks to climate and vegetation change in the Arctic. Documented increases in productivity and above-ground phytomass may be modest compared to potentially vulnerable soil C that could be metabolized as a result of shrub expansion or other biotic and abiotic drivers of change in the circumpolar North. If shrub- and tree-dominated communities continue to expand northwards, then increases in productivity may accelerate C cycling (and release) to a greater extent than any additional sequestration of C. Improved process understanding is required to underpin improvements in Earth System models.

## Supporting Information

**Table S1.** Geographical details of each transect at Abisko and Vassijaure sites. ‘Elevation change’ and ‘heading’ refer to transects from heath to forest plots. Plots on transects run approximately in a straight line.
