# Supplementary material for: Rapid carbon turnover beneath shrub and tree vegetation is associated with low soil carbon stocks at a subarctic treeline
Source: Glob Chang Biol. 2015 Feb 18;21(5):2070–81. doi: 10.1111/gcb.12793 (PMC4657486; doi:10.1111/gcb.12793)
Supplement: Supplementary file 1 — Table S1. Geographical details of each transect at Abisko and Vassijaure sites. ‘Elevation change’ and ‘heading’ refer to transects from heath to forest plots. Plots on transects run approximately in a straight line. [file gcb0021-2070-sd1.docx]

| Abisko | Transect length (m) | Elevation change (m) | Heading (degrees) |
| --- | --- | --- | --- |
| A1 | 74 | +5 | 169 |
| A2 | 70 | -11 | 314 |
| A3 | 87 | +6 | 164 |
| A4 | 71 | -11 | 322 |
| B1 | 82 | -8 | 337 |
| B2 | 97 | +3 | 213 |
| B3 | 77 | +2 | 59 |
| B4 | 52 | -4 | 355 |
| C1 | 59 | -7 | 350 |
| C2 | 53 | -7 | 2 |
| C3 | 60 | +1 | 219 |
| C4 | 31 | -1 | 71 |
| Average | 67.8 | -2.7 |  |
|  |  |  |  |
| Vassijaure |  |  |  |
| 1 | 77 | 4 | 124 |
| 2 | 22 | -12 | 358 |
| 3 | 59 | 1 | 342 |
| 4 | 61 | 2 | 119 |
| 5 | 50 | 0 | 234 |
| 6 | 70 | -8 | 30 |
| 7 | 67 | 4 | 153 |
| Average | 58 | -1.2 |  |

**Supporting information**

**Table S1**: Geographical details of each transect at Abisko and Vassijaure sites. ‘Elevation change’ and ‘heading’ refer to transects from heath to forest plots. Plots on the transects run approximately in a straight line.
